# Immune Mechanisms in Vascular Disease and Stroke

**DOI:** 10.1155/2014/730691

**Published:** 2014-08-14

**Authors:** Ban-Hock Toh, Alexander Bobik, Tin S. Kyaw, Grant R. Drummond, Christopher G. Sobey, Tomasz J. Guzik

**Affiliations:** ^1^Centre for Inflammatory Diseases, Department of Medicine, Southern Clinical School, Faculty of Medicine, Nursing and Health Sciences, Monash University, Clayton, VIC, Australia; ^2^Baker IDI Heart and Diabetes Institute, Melbourne, Australia; ^3^Department of Pharmacology, Faculty of Medicine, Nursing and Health Sciences, Monash University, Clayton, VIC, Australia; ^4^Department of Internal and Agricultural Medicine, Jagiellonian University Medical College, J. Dietl Hospital, Cracow, Poland; ^5^Institute of Cardiovascular and Medical Sciences, University of Glasgow, UK

This special issue is a direct offshoot of a meeting of The Australian-European Consortium on Immune Mechanisms in Vascular Disease and Stroke held on October 10–12, 2013, in Monash University Prato Centre, Prato, Italy, cohosted by Monash University, Australia, and the Faculty of Medicine, Jagiellonian University Medical College, Poland, and organised by Grant Drummond, Chris Sobey, and Tomasz Guzik.

In this special issue, the authors addressed issues related to the immunopathogenesis, diagnosis and therapeutic reversal of atherosclerosis, and the role of the immune system in the development of hypertension and stroke.

In studies of immune activation in atherosclerosis, M. Maddaluno et al. reported that that while murine primary aortic smooth muscle cells express MHC class II and can acquire exogenous antigens, they fail to activate T cells through a failure in antigen presentation and a lack of costimulatory molecule expression. The findings do not support a role of antigen presentation by vascular smooth cells in the activation of T cells in atherosclerosis development. T. Kurita-Ochiai and M. Yamamoto examined the role of* P. gingivalis *or* A. actinomycetemcomitans *in augmenting inflammatory mechanisms and oxidative modification in the formation and activation of atherosclerotic plaques in ApoE-deficient mouse model fed a high-fat diet. They also examined whether mucosal vaccination with a periodontal pathogen or the anti-inflammatory activity of catechins can reduce periodontal pathogen-accelerated atherosclerosis. J. Maciag et al. examined endothelial function and major vascular disease risk factors in patients with dentures with and without clinical and microbiological features of patients' denture-related stomatitis. Patients with denture-related stomatitis had significantly lower flow mediated dilatation suggesting that it is associated with endothelial dysfunction that may predispose to atherosclerosis development. S. Visentin et al. examined relationships between levels of adipocytokine (adiponectin and leptin), markers of inflammation (tumor necrosis factor *α*, interleukin-6, and C reactive protein), and vascular remodelling in pregnancies complicated by intrauterine growth restriction. They found that these foetuses had a higher intima medial thickness that was associated with decreased blood adiponectin levels and increased adipocytokines concentrations that might be related to a greater risk of vascular remodeling.

I. A. Sobenin et al. examined low density lipoprotein-containing circulating immune complexes (LDL-CIC) and found that the titer of LDL-CIC in blood serum significantly correlated with atherosclerosis progression in humans and has the highest diagnostic value among other measured serum lipid parameters. They suggested that elevated CIC-cholesterol might well be a possible risk factor of coronary atherosclerosis.

M. A. Ulleryd et al. reported that metoprolol significantly reduced atherosclerotic plaque area and macrophage content in the thoracic aorta, accompanied by reduced serum levels of TNF*α* and CXCL1 chemokine while total cholesterol levels were not affected. The findings suggest a potential role for metoprolol in the therapeutic management of patients with atherosclerosis.

Q. Dinh et al. reviewed evidence from human and animal studies that inflammation, oxidative stress, and endothelial dysfunction lead to the development of hypertension. Other potential proinflammatory conditions such as aging and elevated aldosterone that contribute to hypertension were also discussed. The evidence suggested that inflammation can lead to the development of hypertension and that oxidative stress and endothelial dysfunction are involved in the inflammatory cascade. The findings point to the potential therapeutic benefit of anti-inflammatory drugs and statins as antihypertensive therapy. C. T. Chan et al. described the protein targets of antibodies that are elevated in individuals with essential and pregnancy-related hypertension and the likely pathophysiological consequences of antibody binding to these targets. Potential mechanisms that underlie elevated antibody levels in hypertensive individuals were discussed and therapeutic opportunities that could arise with a better understanding of how and why antibodies are produced in hypertension were outlined. X. Xu and Y. Jiang reviewed the role of the innate immune system in stroke.

*Ban-Hock Toh* *Alexander Bobik* *Tin S. Kyaw* *Grant R. Drummond* *Christopher G. Sobey* *Tomasz J. Guzik*

